# Efficient Algorithms for Max-Weighted Point Sweep Coverage on Lines

**DOI:** 10.3390/s21041457

**Published:** 2021-02-19

**Authors:** Dieyan Liang, Hong Shen

**Affiliations:** School of Computer Science and Engineering, Sun Yat-Sen University, Guangzhou 510275, China; ldiey@mail2.sysu.edu.cn

**Keywords:** WSN, mobile sensors, sweep coverage, approximation algorithm, combinatorial mathematics

## Abstract

As an important application of wireless sensor networks (WSNs), deployment of mobile sensors to periodically monitor (sweep cover) a set of points of interest (PoIs) arises in various applications, such as environmental monitoring and data collection. For a set of PoIs in an Eulerian graph, the point sweep coverage problem of deploying the fewest sensors to periodically cover a set of PoIs is known to be Non-deterministic Polynomial Hard (NP-hard), even if all sensors have the same velocity. In this paper, we consider the problem of finding the set of PoIs on a line periodically covered by a given set of mobile sensors that has the maximum sum of weight. The problem is first proven NP-hard when sensors are with different velocities in this paper. Optimal and approximate solutions are also presented for sensors with the same and different velocities, respectively. For *M* sensors and *N* PoIs, the optimal algorithm for the case when sensors are with the same velocity runs in O(MN) time; our polynomial-time approximation algorithm for the case when sensors have a constant number of velocities achieves approximation ratio $\frac{1}{2}$; for the general case of arbitrary velocities, $\frac{1}{2\alpha }$ and $\frac{1}{2}\left(1-1/e\right)$ approximation algorithms are presented, respectively, where integer α≥2 is the tradeoff factor between time complexity and approximation ratio.

## 1. Introduction

Coverage is one of the most important applications of wireless sensor networks (WSN), where sensors are placed on an area of interest to monitor the environment and detect extraordinary activities. There have been many studies on this topic across different subject areas including discrete points [1,2], 2-dimensional surfaces [3], 3-dimensional surfaces [4], 3-dimensional spaces, fences [5,6], and so on. Based on sensors’ characteristic, special factors should be considered, such as energy efficiency, maintaining connectivity, and so on [7,8,9,10].

However, most of the existing works have mainly focused on continuous coverage, where sensors stay still after begin placed on their objective locations. Only recently has sweep coverage been brought up for the periodical coverage situation, which arises in many applications. For example, guards need to patrol a barrier periodically according to the time needed for intruders to cross it; Information collectors should collect information from the objective sensors periodically to avoid their memory overflow. In those situations, the objects do not need to be covered continuously. So, mobile sensors can move around to cover more objects than in the continuous coverage situation to reduce monitoring cost. For its cost effectiveness, sweep coverage has attracted increasing attention [11,12,13,14,15].

**Figure 1:**
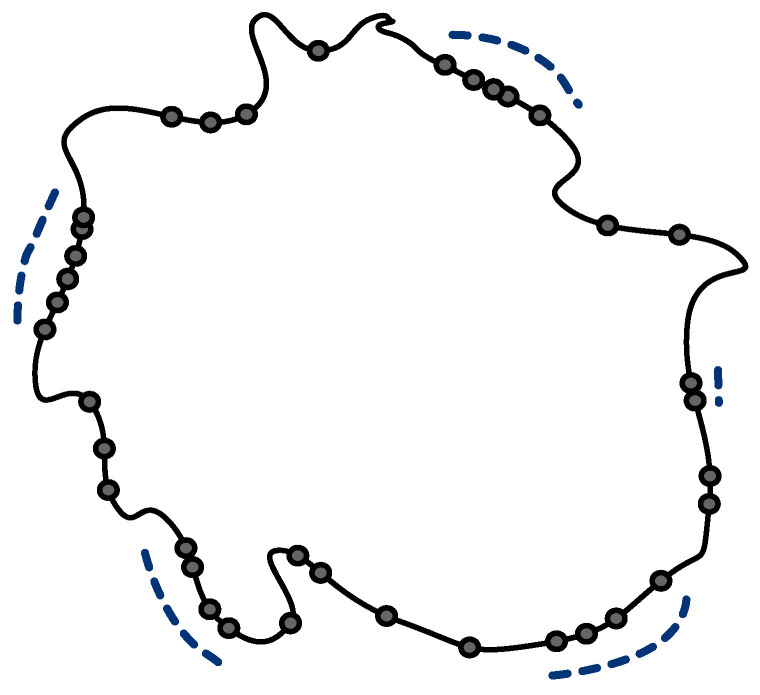
Mobile sensors sweep coverage around island to collect the data from the static sensors.

Point sweep coverage was first brought up in Reference [11], in which the authors introduced the problem of deploying the minimum number of mobile sensors to cover a given set of points of interest (PoIs) in the Euclidean space. The problem was shown Non-deterministic Polynomial Hard (NP-hard), and could not be approximated within the factor of 2, even for the case that all sensors are with the same velocity. In practice, with the limited energy, the velocity of a mobile sensor decreases as energy consumption increases. It is more realistic to consider the general case that mobile sensors are with arbitrary velocities. However, in Reference [16], the authors proved that no polynomial-time constant-factor approximation algorithm exists to solve this problem for mobile sensors with different velocities unless P=NP. In this paper, we study a variant of this problem, named Max-weighted Point Sweep Coverage, to  find the set of PoIs that have the maximum sum of weight, periodically covered by a given set of sensors with different velocities. We show that it is NP-hard even when the PoIs are distributed on a line. In applications, since resources are always limited, it is desirable to use a given set of sensors to cover as many PoIs as possible. The Max-weighted Point Sweep Coverage problem aims at maximizing the utilization of the given mobile sensors for addressing these application requirements. In addition, PoIs located on a line is a common scenario in many applications. For example, as illustrated in Figure 1, for ocean information stored in static sensors placed at key locations along a line, we need to deploy a set of mobile sensors to collect the data from the static sensors periodically to avoid static sensors memory overflow. Other applications can be found in security, forest conservation, resource exploration, and so forth. Therefore, we focus on the Max-weighed Point Sweep Coverage on the line (MPSCL) problem and discuss the proper polynomial-time algorithms in different cases.

In this paper, we define the MPSCL problem, prove that it is NP-hard by showing that a special case of its decision version is NP-complete (NPC), and present optimal and approximation algorithms for the cases of mobile sensors with the same and different velocities, respectively.

The main contributions of this paper are summarized as follows:

- We define the MPSCL problem and prove it is NP-hard through a reduction from the 3-Partition problem.
- For the special cases of the MPSCL problem when sensors have the same velocity, we present an optimal algorithm applying dynamic programming.
- For the special cases of the MPSCL problem when sensors have a constant number of velocities, we present a $\frac{1}{2}$-approximation algorithm by extending the solution for the same-velocity case.
- For the general cases of the MPSCL problem when sensors have arbitrary velocities, we propose three approximation algorithms. One achieves approximation ratio $\frac{1}{2\alpha }$ by velocity rounding, where integer α≥2 is the tradeoff factor between time complexity and approximation ratio. The second and third one are, respectively, a random and a deterministic $\frac{1}{2}\left(1-1/e\right)$ approximation algorithm by randomized rounding and derandomized technique.

The rest of this paper is organized as follows: Section 2 describes some related work. In Section 3, the definition and NP-hardness proof of the MPSCL problem are given. In Section 4, we present our optimal and approximation algorithms for different cases of the MPSCL problem. Section 5 presents the simulation results and investigates the performance of the algorithms. Section 6 concludes the paper.

## 2. Related Work

The point sweep coverage was firstly brought up in Reference [11]. The authors presented the Min-Sensor Sweep Coverage problem (MSSC) to find the minimum number of sensors to sweep cover PoIs in Eulerian graph, which was proven NP-hard by transforming the Traveling Salesman Problem (TSP) to it when the mobile sensors were with the same velocity. The problem could not be approximated within ratio 2 and local algorithms could not work. In Reference [17], the authors distinguished sensors’ strategies, proposed MinExpand algorithm for un-cooperated sensors and Osweep algorithm for cooperated sensors, respectively. A mistake of approximation analysis of prior papers was rectified by Gorain et al. [16], in which a 3-approximation algorithm for the MSSC problem was proposed and it was still the best approximation algorithm for MSSC till now. Non-existence of polynomial-time constant-factor approximation algorithms for MSSC when sensors were with different velocities was also proven. Some variants of MSSC were presented. When PoIs had different sweep periods, an O(logρ)-approximation algorithm was proposed, where ρ was the ratio of the maximum and minimum sweep periods among PoIs. The area sweep coverage problem and line sweep coverage problem were proposed and shown NP-hard, and approximation algorithms of ratio $\left(\sqrt{2}+\frac{2-\sqrt{2}}{mn}\right)$ and 2 were proposed, respectively [18,19]. In Reference [20], a variation of the MSSC problem called the DistanceSensitive-Route-Scheduling problem was studied, where the impact of sensing range was taken into account. The impact of sensing range in the sweep coverage problems shortened the trajectory length of mobile sensors to reduce needed sensors. In Reference [21], the authors assumed the consumption of energy was different between mobile sensors and static sensors, and proposed two variations of the MSSC problem. One was the energy efficient sweep coverage problem to minimize the total energy consumption in every unit of time, which could not be approximated within a factor of 2, and an 8-approximation algorithm for that was proposed. Another was the energy restricted min-sensor sweep coverage problem, for which a $\left(5+\frac{2}{\alpha }\right)$-approximation algorithm was proposed. Gorain et al. took barrier sweep coverage into account [22]. They presented a energy restricted barrier sweep coverage problem and proposed $\frac{13}{3}$ approximation algorithm.

The concept of sweep coverage appeared in the contexts of robotics concerned sweep covering continuous lines, and the problem was called boundary patrolling or fence patrolling. In these contexts, the mobile sensors might be with different velocities and the aim was to find the minimum idleness, i.e., the longest time interval during which there was at least one point on the boundary uncovered by any mobile sensors. In Reference [23], the authors firstly studied boundary patrolling problem and proposed two intuitive algorithms for open and close fence patrolling. The optimality of these algorithms was disproved in Reference [24,25,26], in which the examples were proposed to illustrate that the idleness could be reduced to 41/42, 24/25, and even 3/4 by special design, assuming the idleness of proportional solution presented in Reference [23] was 1. Even though the optimality of algorithms presented by Czyzowicz et al. was disproved, the optimal solution had not been brought up yet. In Reference [27], the authors extended the scenes of the min-idleness sweep coverage problem to chains, trees, and cyclic roadmaps when the mobile sensors were identical. Within tolerance ϵ, they could get an optimal idleness when PoIs were on a chain in time complexity $O(nlog\left(\epsilon^{-1}\right))$. And an 8-approximation algorithm was proposed to find min-idleness when PoIs were on cyclic roadmap for which the problem was NP-hard. In Reference [28], the authors described a fragmented boundary environment and found the optimal patrolling for min-idleness in that environment when the sensors were with the same velocity. Min idleness problem was also called as min-period problem. Gao et al. studied Min-peroid Sweep Coverage (MPSC) problem when the sensors did not cooperate with each other [29], in which, they proposed a nearly 5-appr algorithm for MPSC when the sensors with the same velocity covered target points on 2-D plane and a 5α-appr algorithm when the sensors have different velocities, where α was the ratio of the maximum velocity to the minimum one. They also had extended it to the scene where a graph needed to be covered. In Reference [30], Gao et al. studied Cooperative Sweep Coverage (CSC) problem, when the sensors with the same velocity, and proposed a 4-appr algorithm for general case and an optimal solution for the special case when PoIs were on a line. They also considered the situation in which each track cycle must cover at least one sink.

## 3. Preliminary and Problem Statement

In this section, we will study the Max-weighted Point Sweep Coverage on Lines (MPSCL) problem. We first give its definition and then prove it is NP-hard.

**Definition** **1.** *(Max-weighted Point Sweep Coverage on Lines) Given a set of M mobile sensors $S=\{s_{1},s_{2},..,s_{M}\}$ with velocities $V=\{v_{1},v_{2},\ldots ,v_{M}\}$ and a set of PoIs $P=\{p_{1},p_{2},..,p_{N}\}$ distributed on a line, where each $p_{i}$ needs to be monitored every time period T, i.e., T-sweep covered, find a set of PoIs of maximum summed weight that is T-sweep covered by the given set of mobile sensors.*

Note that static sensors are regarded as special mobile sensors with velocity 0. For simplicity, we sometimes say “cover” or “sweep cover” to replace “*T*-sweep cover”.

In the existing work, two kinds of strategies are proposed, separation strategy [28] and cooperation strategy [27], respectively. Their definitions are below:

**Definition** **2.** *(separation strategy) Mobile sensors move back and forth on the line segment to sweep cover PoIs without cooperating with others, where each PoI would meet the same sensor within time period T.*

**Definition** **3.** *(cooperation strategy) Sensors cooperate with others to cover the same line segment, i.e., the PoIs on the line segment would meet another sensor within T time after covered by one sensor last time.*

Under separation strategy, every sensor $s_{i}\in S$ has its own coverage $r_{i}=v_{i}T/2$. So, a separation strategy can be expressed by the first PoI’s location of every sensor’s coverage. Wthout loss of generality, we assume that the set of PoIs are located on the *x*-axis at coordinates $X=\left\{x_{1}=0,x_{2},\ldots ,x_{N}\right\}\left(x_{1}<x_{2}<\cdots <x_{N}\right)$, and the coordinate of PoI *l* is $x_{l}$. The deployment of sensor $s_{i}$ can be described as $\left[x_{l},x_{l}+v_{i}T/2\right]$, l∈[1,N].

For cooperation strategy, the path of each sensor is a periodical folded line, which is more complicated to be expressed than the separation strategy. Separation strategy may not result in an optimal solution. Some examples show that separation strategy may be slightly worse than cooperation strategy in some special cases, i.e., the covering range covered by a set of mobile sensors under separation strategy is slightly shorter [24,25]. For example, in Reference [25], six sensors with velocities {1,1,1,1,7/3,1/2} can 1-sweep cover PoIs on a line segment of length 7/2 under a cooperation strategy, which is longer than that of separation strategy, (1+1+1+1+7/3+1/2)/2=41/12. However, compared to the complication of coorperation strategy, separation strategy is easier to be analyzed.

The notations are summarized in Table 1.

### Problem Hardness

In this section, we present the definition of a special case of the MPSCL problem when PoIs’ weight is the same as Definition 4. By proving that it is NP-complete, we show the MPSCL problem is NP-hard.

**Definition** **4.** *(Point Sweep Coverage on Lines) Given a set P of PoIs on lines, a set S of mobile sensors, and a positive integer K≤|P|, is there a strategy for sensors S such that no less than K PoIs are sweep covered?*

Our NP-completeness proof is based on a reduction of the following 3-Partition problem, which is well-known NP-complete to the Point Sweep Coverage on Line (PSCL) problem.

**Definition** **5.** *(3-Partition) [31]. INSTANCE: Set A of 3m elements, a bound $B\in Z^{+}$, and a size $s\left(a\right)\in Z^{+}$ for each a∈A such that B/4<s(a)<B/2 and such that $\sum_{a\in A}s\left(a\right)=mB.$ QUESTION: Can A be partitioned into m disjoint sets $A_{1},A_{2},\ldots ,A_{m}$ such that, for 1≤k≤m*, *$\sum_{a\in A_{k}}s\left(a\right)=B$ (note that each $A_{i}$ must therefore contain exactly three elements from A)?*

**Theorem** **1.** *Point Sweep Coverage on Lines problem is NP-complete.*

**Proof.**  Given a 3-Partition instance, like Definition 5, we construct a MPSCL instance. Given a set of *N* PoIs with their positions $\left\{x_{1},x_{2},\ldots ,x_{N}\right\}\left(x_{1}<x_{2}<\cdots <x_{N}\right)$, N=(2B+2)×m. The positions of PoIs satisfy the equation below, where $d_{i}=x_{i+1}-x_{i}\left(1\leq i<N\right)$ means the distance between the (i+1)th PoI and *i*th PoI.

(1) $$\begin{array}{cc} x_{1} & =0\\ d_{i} & =B, \end{array}$$

(2) $$\begin{array}{cc} & \left(i=k\times (2B+2);1\leq k\leq m-1\right)\\ d_{i} & =B/(2B+1), \end{array}$$

(3) $$\begin{array}{cc} & ((k-1)\times (2B+2)+1\leq i<k\times (2B+2);1\leq k\leq m). \end{array}$$

Given a set S of M=3m mobile sensors, the velocity of mobile sensor $s_{j}$ is $v_{j}=2\times s\left(a_{j}\right)/T$ for $a_{j}\in A$(1≤j≤M). As mentioned before, if under separation strategy, each mobile sensor has its own covering range $r_{j}=v_{j}T/2=s\left(a_{j}\right)$, 1≤j≤M. Then, $B/4<r_{j}<B/2$, $\sum_{1\leq j\leq M}r_{j}=mB.$

The object of the PSCL instance is to cover at least K=N PoIs.

Let $B_{k}$ denote the line segment from $x_{(k-1)\times (2B+2)+1}$ to $x_{k\times (2B+2)}$ for 1≤k≤m. Because of Equation (3), we get $|B_{k}|=B$. If the 3-Partition instance is satisfied, we get $\sum_{a\in A_{k}}s\left(a\right)=B$, where each $A_{k}$ contains exactly three elements from *A*. It means, if we can obtain proper 3 mobile sensors to sweep cover each $B_{k}$ for 1≤k≤m, then we get a proper deployment for the PSCL problem.

Conversely, for the other side, if we have an unique solution for the PSCL problem, because of Equation (2), mobile sensors $s_{j}$ (1≤j≤M) must cover some part of line segment $B_{k}$(1≤k≤m) since the gap between $B_{k}$ and $B_{k^{\prime }}$$\left(k\neq k^{\prime }\right)$ is too big. Because $B/4<r_{j}<B/2$ and $r_{j}\in Z^{+}$ for 1≤j≤M, more than 2 sensors are needed to cover each segment $B_{k}$(1≤k≤m). Considering there are 3m sensors for *m* line segments $B_{k}\left(1\leq k\leq m\right)$, we get exactly 3 mobile sensors to cover each segment $B_{k}$(1≤k≤m). The separation strategy is optimal for three mobile sensors [25]. Thus, for arbitrary $B_{k}$(1≤k≤m), assuming covering ranges of the 3 mobile sensors are $r_{j1},r_{j2},r_{j3}$, respectively, the best total covering range of the 3 mobile sensors is

$$r_{j1}+r_{j2}+r_{j3}\geq B-2B/\left(2B+1\right).$$

Otherwise, the three sensors are not enough to cover all the PoIs on $B_{k}$. And because $r_{j1},r_{j2},r_{j3}\in Z^{+}$, we get $r_{j1}+r_{j2}+r_{j3}\geq B$. If $r_{j1}+r_{j2}+r_{j3}>B$, there must exist $r_{j^{\prime }1}+r_{j^{\prime }2}+r_{j^{\prime }3}<B\left(j^{\prime }\neq j\right)$, making a contradiction. So, $r_{j1}+r_{j2}+r_{j3}=B$, satisfying the solution to the 3-Partition problem.

**Figure 2:**
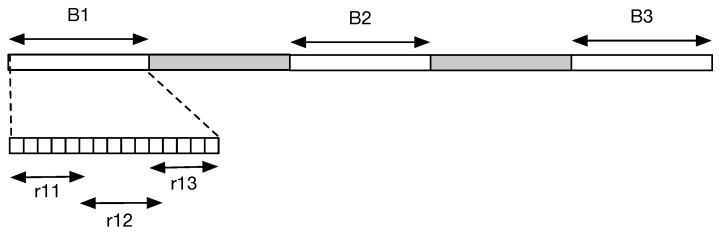
The instance of Point Sweep Coverage on Line (PSCL) when m=3, B=7, K=48. It contains 48 points of interest (PoIs) and 9 sensors. The PoIs are distributed on subsegment $B_{j}$ (1≤j≤3). When the covering range of sensor $s_{i}$ is $\frac{7}{4}\leq r_{i}\leq \frac{7}{2}$ (1≤i≤9), the proper deployment is to deploy 3 sensors to each $B_{j}$.

Note that the 3-Partition problem is strongly an NPC problem, which means it is also NPC even if *B* is bounded by polynomial in *m*. So, when *B* is bounded by polynomial in *m*, the instance of PSCL can be constructed from an arbitrary 3-Partition instance in polynomial time. Now, we have shown the reduction from the 3-Partition problem to the PSCL problem. An example is illustrated in Figure 2.

Given the strategies of sensors, it is easy to check if or not there are *K* PoIs are in the coverage of the sensors in polynominal time. Thus, the decision version of the PSCL problem is in NPC.

The theorem is proven.    □

Hence, we have:

**Corollary** **1.** *The Max-weighted Point Sweep Coverage Problem is NP-hard.*

## 4. Algorithms

In this section, we present an optimal solution for the special case of sensors with same velocity and approximation solutions for sensors with different velocities, respectively. We assume that the number of PoIs is greater than that of mobile sensors, i.e., N>M; otherwise, the problem has a trivial solution.

### 4.1. Optimal Solution for Sensors with Same Velocity

In this subsection, we show that there is a polynomial-time optimal solution for MPSCL when sensors have the same velocity.

Reference [27] showed that separation strategy can yield an optimal solution when PoIs on the line are sweep covered by mobile sensors with the same velocity as Theorem 2 because, when two sensors in opposite directions meet each other, they can “exchange” roles.

**Theorem** **2.** *Separation strategy yields an optimal solution for the Max-weight Sweep Coverage on Lines problem if the given set of mobile sensors have the same velocity.*

In our algorithm, we first apply a dynamic programming algorithm to find the optimal separation strategy of MPSCL. According to Theorem 2, it is also an optimal solution for MPSCL. In the separation strategy, sensors have the same size of coverage with the same velocity *v*. So, in our algorithm, we can first judge whether the given set of mobile sensors can sweep cover all the PoIs by examining the coverage from the 1st to the *N*th PoI without overlapping. If “yes”, the given set of mobile sensors can sweep cover all the PoIs and the maximum weight is the summed weight of all the PoIs. Otherwise, we apply dynamic programming to obtain the optimal solution as follows.

Let OPT(i,j) denote the maximum summed weight of the PoIs covered by *i* sensors from the *j*th to PoI *N*. $n_{j}$ denotes the number of PoIs covered by one sensor from the *j*th PoI, i.e., the number of PoIs located in coordinate interval $\left[x_{j},x_{j}+vT/2\right]$, and $\omega_{j}$ is the summed weight of the $n_{j}$ PoIs. The recursive formulation of OPT(i,j) is given below:

$$OPT\left(i,j\right)=\begin{array}{c} max\left\{\begin{array}{c} OPT(i,j+1),\\ OPT\left(i-1,j+n_{j}\right)+\omega_{{}_{j}} \end{array}\right\},\;\;1\leq i\leq M,1\leq j<N-1, \end{array}$$

with boundary conditions

$$\begin{array}{c} \left\{\begin{array}{cc} OPT(0,j)=0 & 0\leq j\leq \;N\\ OPT(i,N)=1 & 1\leq i\leq M \end{array}\right.. \end{array}$$

The first step of our algorithm, deciding whether all the PoIs can be covered, takes O(N) time. The time complexity of the dynamic programming is O(MN). Thus, the time complexity of the optimal algorithm for MPSCL with the same velocity is O(MN). The algorithm is straightforward; hence, its description is omitted.

### 4.2. $\frac{1}{2}$-Approximation Solution for Sensors with a Constant Nunber of Velocities

Now, we discuss the MPSCL problem when sensors have *K* different velocities, called *K*-velocity MPSCL, where *K* is a constant. In fact, it is not hard to see that the uniform velocity case of the MPSCL problem is a special case of *K*-velocity MPSCL that K=1. The only difference is that separation strategy is not an optimal strategy any more when K≥2 and M≥4 [25]. However, it is easy to find the set of PoIs covered with the maximum summed weight under separation strategy since it has the nature of optimal substructure. So, we use the dynamic programming method to find an optimal separation strategy and prove it is a $\frac{1}{2}$-approximation algorithm for the *K*-velocity MPSCL problem considering the difference between the separation strategy and cooperation strategy.

Let $m_{i}$ be the number of sensors with velocity $v_{i}$, then $M=\sum_{i=1}^{K}m_{i}$. $OPT_{S}\left(i_{1},i_{2},\ldots ,i_{K},j\right)$ denotes the maximum summed weight of the covered PoIs when there are $\sum_{h=1}^{K}i_{h}$ sensors to cover the line segment from the PoI *j* to PoI *N* under separation strategy, where $i_{h}$ is the number of sensors with velocity $v_{h}$(1≤h≤K). Denote the number of PoIs covered by a sensor with velocity $v_{i}$ from the PoI *j* by $n_{ij}$, i.e., the number of PoIs located in coordinate interval $\left[x_{j},x_{j}+v_{i}T/2\right]$, and the summed weight of the $n_{ij}$ PoIs by $\omega_{ij}$. Below is the recursive formulation of the solution:

$$OPT_{S}\left(i_{1},i_{2},\ldots ,i_{K},j\right)=max\left\{\begin{array}{c} OPT_{S}\left(i_{1},i_{2},\ldots ,i_{K},j+1\right),\\ OPT_{S}\left(i_{1}-1,i_{2},\ldots ,i_{K},j+n_{1j}\right)+\omega_{1j},\\ OPT_{S}\left(i_{1},i_{2}-1,\ldots ,i_{K},j+n_{2j}\right)+\omega_{2j},\\ \ldots ,\\ OPT_{S}\left(i_{1},i_{2},\ldots ,i_{K}-1,j+n_{Kj}\right)+\omega_{kj} \end{array}\right\},\begin{array}{c} 1\leq i_{h}\leq m_{h}\;\&\\ 1\leq h\leq K\;\&\\ 1\leq j\leq (N-1) \end{array}.$$

The boundary conditions are

$$\left\{\begin{array}{cc} OPT_{S}\left(0,0,\ldots ,0,j\right)=0\; & \;\;\;\;0\leq j\leq \;N\\ OPT_{S}\left(i_{1},i_{2},\ldots ,i_{K},N\right)=1\; & 0\leq i_{h}\leq m_{h},1\leq h\leq K,\sum_{h=1}^{K}i_{h}\geq 1 \end{array}\right..$$

In Algorithm 1, we maintain a (K+1)-dimensional table Tb to record the optimal value $OPT_{S}\left(i_{1},i_{2},\ldots ,i_{K},j\right)$ for $i_{h}\in \left[0,m_{h}\right]$ and 1≤h≤K, 1≤j≤N. By tracing back the information of table Tb, we can obtain the optimal separation strategy, in which every sensor’s location is recored in an array entry Lc(h) for h=1,…,K. Each entry Lc(h) is a list of $m_{h}$ locations for the $m_{h}$ sensors with velocity $v_{h}$. We say the location of a sensor is the location of the first PoI in the its coverage. In separation strategy, given the velocity $v_{i}$ and its location, the deployment of sensor $s_{i}$ is set.

In Algorithm 1, it takes $O(N\times \prod_{i}\left(m_{i}+1\right))$ time to construct table Tb, and takes O(N×K) time to trace back to get the optimal solution. $N\times \prod_{i}\left(m_{i}+1\right)\leq N\times {\left(M/K+1\right)}^{K}$, so the time complexity of Algorithm 1 is $O(N\times {\left(M/K+1\right)}^{K})$. Because *K* is a constant integer, the algorithm is polynomial time algorithm. Now, we show that Algorithm 1 is an $\frac{1}{2}$-approximation algorithm for the *K*-velocity MPSCL problem. Before that, we give a more general theorem, showing that the β-approximation algorithm for the optimal separation strategy of MPSCL can yield a $\frac{\beta }{2}$-approximation algorithm for MPSCL.

**Theorem** **3.** *A β-approximation algorithm for the optimal separation strategy of MPSCL can be turned to be a $\frac{\beta }{2}$-approximation algorithm for MPSCL, where β≤1.*

**Proof.**  Denote by *A* the β-approximation algorithm for the optimal separation strategy of MPSCL, and by $A_{o}$ the optimal algorithm for MPSCL. W.l.o.g., assume $A_{o}$ covers two sets of line segments $L_{1}$ and $L_{2}$ by the separation strategy and cooperation strategy, respectively.

For $L_{1}$, clearly the sum of weight of the covered PoIs by $A_{o}$, $OPT_{1}$, cannot exceed that covered by the optimal separation strategy, $OPT_{1}^{S}$, and hence 1/β of that by *A*, $APPROX_{1}^{S}$, using the same set of sensors: $OPT_{1}\leq OPT_{1}^{S}=\frac{1}{\beta }\times APPROX_{1}^{S}\Rightarrow APPROX_{1}^{S}\geq \beta \times OPT_{1}$.

For $L_{2}$, because the coverage $r(S^{\prime })$ of any set of sensors $S^{\prime }\subseteq S$ by the cooperation strategy is bounded by $\sum_{i=1}^{|S^{\prime }|}v_{i}T$, which is twice of the coverage by the separation strategy, the sum of weight of the covered PoIs by $A_{o}$, $OPT_{2}$, cannot exceed twice of the optimal separation strategy, $OPT_{2}^{S}$, and hence 2/β of that by *A*,$APPROX_{2}^{S}$, using the same set of sensors: $OPT_{2}\leq 2*OPT_{2}^{S}=\frac{2}{\beta }\times APPROX_{2}^{S}\Rightarrow APPROX_{2}^{S}\geq \frac{\beta }{2}\times OPT_{2}$.

Summing up the above immediately yields the $\frac{\beta }{2}$ approximation ratio of algorithm *A* to the optimal algorithm for MPSCL.    □

**Theorem** **4.** *Algorithm 1 is a $\frac{1}{2}$-approximation algorithm for the K-velocity MPSCL problem.*

**Proof.**  Algorithm 1 is for the optimal separation strategy of the *K*-velocity MPSCL. According to Theorem 3, Algorithm 1 is a $\frac{1}{2}$-approximation algorithm for the *K*-velocity MPSCL problem.    □

**Algorithm 1** MPSCL-K-Velocities**Input:** A set of sensors S with velocities $V=\{v_{1},v_{2},\ldots ,v_{M}\}$, A set of PoI P with locations $X=\{x_{1},x_{2},\ldots ,x_{N}\}$ and weight $W=\{\omega_{1},\omega_{2},\ldots ,\omega_{N}\}$, sweep period *T*, locations ${}^{*}Lc$.
**Output:** The sweep covered set $P_{S}$ of PoIs.
1: //initialization
2: group the velocities V into *K* different velocities $\left\{{\bar{v}}_{1},{\bar{v}}_{2},\ldots ,{\bar{v}}_{K}\right\}$
3: let $m_{i}$ is the number of sensors with velocity ${\bar{v}}_{i}$
4: **for**
i←1 to *K*
**do**
5: **for**
j←N to 1 **do**
6:  count the number $n_{ij}$ of PoIs covered by a sensor with velocity $V_{i}$ and location $x_{j}$
7:  let $\omega_{ij}$ be the sum of weight of the $n_{ij}$ PoIs;
8: **end for**
9: **end for**
10: //dynamic programming to obtain the maximum summed weight
11: initial table Tb according to boundary conditions
12: **for**
j←N to 1 **do**
13: **for**
$i_{1}\leftarrow 1$ to $m_{1}$
**do**
14:  **for**
$i_{2}\leftarrow 1$ to $m_{2}$
**do**
15:   **for** … **do**
16:    **for**
$i_{K}\leftarrow 1$ to $m_{K}$
**do**
17:     call the recursive formulation to calculate $OPT_{S}\left(i_{1},i_{2},\ldots ,i_{K},j\right)$, recorded in Tb
18:    **end for**
19:   **end for**
20:  **end for**
21: **end for**
22: **end for**
23: // tracing back to obtain the optimal solution
24: set $i_{1}=m_{1},i_{2}=m_{2},\ldots ,i_{K}=m_{K}$
25: initial array entry Lc
26: **for**
j←1 to N−1
**do**
27: **if**
$OPT_{S}\left(i_{1},i_{2},\ldots ,i_{K},j\right)!=OPT_{S}\left(i_{1},i_{2},\ldots ,i_{K},j+1\right)$
**then**
28:  **for**
h←1 to *K*
**do**
29:   **if**
$i_{h}>0$
&&
$OPT_{S}\left(i_{1},i_{2},\ldots ,i_{h},\ldots ,i_{K},j\right)==O\left(i_{1},i_{2},\ldots ,i_{h}-1,\ldots ,i_{K},j+n_{i_{h}j}\right)+\omega_{i_{h}j}$
**then**
30:    add $x_{j}$ to Lc(h); $j+=n_{i_{h}j}$
31:    $i_{h}--$
32:   **end if**
33:  **end for**
34: **end if**
35: **end for**
36: **if** exist any sensor with velocity ${\bar{v}}_{i}$ unoccupied **then**
37: add $x_{N}$ to Lc(i)
38: **end if**
39: place sensors to their corresponding locations in Lc, the union set of covered PoIs is $P_{S}$
40: **return**
$P_{S}$

### 4.3. Approximation Solutions for the General Case of Arbitratry-Velocity Sensors

In this subsection, we discuss MPSCL for the general case, i.e., when sensors have arbitrary velocities and propose three methods. The first method uses rounding and dynamic programming technique and yields a $\frac{1}{2\alpha }$-approximation scheme solution, where integer α≥2. The second uses linear programming relaxation and randomization technique to yield a randomized $\frac{1}{2}\left(1-1/e\right)$-approximation algorithm. Applying the conditional expectations method, we can get the third one, a deterministic $\frac{1}{2}\left(1-1/e\right)$-approximation algorithm by derandomization.

#### 4.3.1. Dynamic-Programming Solution with Velocity Rounding

In the previous subsection, we know Algorithm 1 is the optimal algorithm for the separation strategy of the *K*-velocity MPSCL problem and takes $O(N\times {\left(M/K+1\right)}^{K})$ time. In the general case, *M* sensors would have *M* different velocities, i.e., K=M. Thus, Algorithm 1 would find the optimal separation strategy within the time complexity $O(N*2^{M})$, which is too high. So, we use the rounding method to reduce the number of velocities in order to reduce the time complexity.

In Algorithm 2, for a given set of sensor S with velocities $V=\{v_{1},v_{2},\ldots ,v_{M}\}$, we first round the velocities V to $V^{\prime }=\left\{v_{1}^{\prime },v_{2}^{\prime },\ldots ,v_{M}^{\prime }\right\}$ by applying the formula (4), which contains K≪M different velocities. Then, we run Algorithm 1 on a set of *M* sensors S with rounded velocities $V^{\prime }$ to obtain their locations. Finally, we shift the locations of sensors to avoid their coverage overlapping. That is, since $v_{i}\geq v_{i}^{\prime }$ for 1≤i≤M, there may be overlap between two sensors’ coverage. If so, move the right sensor a minimum distance toward right so that it covers from the next PoI. The shift would increase the number of PoIs covered and the summed weight without changing the approximation ratio.
**Algorithm 2** MPSCL-Velocity-Rounding**Input:** A set of sensors S with velocities $V=\{v_{1},v_{2},\ldots ,v_{M}\}$, A set of PoI P with locations $X=\{x_{1},x_{2},\ldots ,x_{N}\}$ and weight $W=\{\omega_{1},\omega_{2},\ldots ,\omega_{N}\}$, sweep period *T*.
**Output:** The sweep covered set $P_{S}$ of PoIs.
1: set $d_{\min}^{\left(\alpha \right)}=x_{\alpha +1}-x_{1}$;
2: **for**
i←2 to N−α
**do**
3: $d_{\min}^{\left(\alpha \right)}=min\left\{d_{\min}^{\left(\alpha \right)},x_{i+\alpha }-x_{i}\right\}$;
4: **end for**
5: set $v_{d}=max\left\{v_{\min},\frac{d_{\min}^{\left(\alpha \right)}}{2T}\right\}$;
6: **for**
i←1 to *M*
**do**
7: **if**
$v_{i}\geq v_{d}$
**then**
8:  // For the sensors with velocities no less than $v_{d}$
9:  set $v_{i}^{\prime }=\alpha^{\left\lfloor log_{\alpha }\left(v_{i}/v_{d}\right)\right\rfloor }\times v_{d}$;
10: **else**
11:  //For the sensors with velocities less than $v_{d}$
12:  $v_{i}^{\prime }=0$
13: **end if**
14: **end for**
15: set $V^{\prime }=\left\{v_{i}^{\prime }|1\leq i\leq M\right\}$
16: set $K=\lfloor log_{\alpha }\left(v_{max}/v_{d}\right)\rfloor +2$
17: initial an entry array Lc(j) for 1≤j≤K
18: Run Algorithm 1 on sensors S with velocities $V^{\prime }$ and get their locations Lc
19: adjust the coverage of sensors S to avoid overlapping, the union set of PoI covered is $P_{S}$
20: **return**
$P_{S}$

In the rounding step, we round $v_{i}\in V$ to $v_{i}^{\prime }\in V^{\prime }$ as follows. Let $v_{\max}$ and $v_{\min}$ be the largest and smallest non-zero velocities in V, $v_{d}=\max\left\{\frac{d_{\min}^{\left(\alpha \right)}}{2T},v_{\min}\right\}$, where integer α≥2 and $d_{\min}^{\left(\alpha \right)}$ is the minimum distance among every segment of α+1 PoIs, i.e., $d_{\min}^{\left(\alpha \right)}=\min_{1\leq j\leq N-\alpha }\left\{x_{j+\alpha }-x_{j}\right\}$; namely, there are no more than α PoIs on a segment with length less than $d_{\min}^{\left(\alpha \right)}$. We round a group of velocities $v_{i}\in V$ in some interval to the same velocity $v_{i}^{\prime }$ by applying the following mapping:

(4) $$\left\{\begin{array}{cc} v_{i}^{\prime }=0 & \mathrm{for}\;v_{i}<v_{d},\\ v_{i}^{\prime }=\alpha^{j}v_{d} & \mathrm{for}\;\alpha^{j}v_{d}\leq v_{i}<\alpha^{j+1}v_{d},j=0,\;1,\;2,\ldots ,\;\left\lfloor \log_{\alpha }\left(v_{\max}/v_{d}\right)\right\rfloor . \end{array}\right.$$

Clearly, the above mapping rounds all $v_{i}\in \left[\alpha^{j}v_{d},\;\alpha^{j+1}v_{d}\right)$ to $v_{i}^{\prime }=\alpha^{j}v_{d}$ for $0\leq j\leq \lfloor \log_{\alpha }\left(v_{\max}/v_{d}\right)\rfloor$, and all $v_{i}\in \left[0,\;v_{d}\right)$ to $v_{i}^{\prime }=0$. This effectively reduces the number of velocities from *M* to $K=\lfloor \log{}_{\alpha }\left(v_{\max}/v_{d}\right)\rfloor +2\ll M$. By setting the rounding parameter α, we can achieve any desired value of *K* accordingly so as to obtain the algorithm with desired approximation ratio and time complexity.

Now, we prove the performance guarantee of Algorithm 2.

**Theorem** **5.** *Algorithm 2 is a $\frac{1}{2\alpha }$-approximation algorithm for MPSCL, where integer α≥2.*

**Proof.**  We show that the summed weight of the output of Algorithm 2, $A_{2}\left(S\right)$, is $\frac{1}{\alpha }$ of that of Algorithm 1, $A_{1}\left(S\right)$, which is the optimal separation strategy for sensors S. Then, according to Theorem 3, that Algorithm 2 is a $\frac{1}{2\alpha }$-approximation algorithm for MPSCL.

For two sets of sensors, S with velocities $V=\{v_{1},v_{2},\ldots ,v_{M}\}$ and $S^{\prime }$ with velocities $V^{\prime }=\left\{v_{1}^{\prime },v_{2}^{\prime },\ldots ,v_{M}^{\prime }\right\}$ following the mapping according to Equation (4). We need to show that $A_{1}\left(S\right)$ can be covered by α copies of sensors $S^{\prime }$; hence, one copy of sensor $S^{\prime }$ can sweep cover PoIs with weight more than $A_{1}\left(S\right)/\alpha$, i.e., $A_{1}\left(S^{\prime }\right)\geq \frac{1}{\alpha }A_{1}\left(S\right)$.

For sensor $s_{i}\in S$ in velocity range $\alpha^{j}v_{d}\leq v_{i}<\alpha^{j+1}v_{d}$ is mapped to sensor $s_{i}^{\prime }\in S^{\prime }$ with velocity $v_{i}^{\prime }=\alpha^{j}v_{d}$ according to Equation (4). $v_{i}\in \left[v_{i}^{\prime },\alpha v_{i}^{\prime }\right)$; thus, the coverage of $s_{i}$, $R(s_{i})$ can be covered by deploying α copies of sensors $s_{i}^{\prime }$ with velocities $v_{i}^{\prime }$. For sensor $s_{i}\in S$ with velocity $v_{i}<v_{d}$ is mapped to sensor $s_{i}^{\prime }\in S^{\prime }$ with velocity $v_{i}^{\prime }=0$. Note that $v_{d}=\max\left\{\frac{1}{2T}d_{\min}^{\left(\alpha \right)},v_{\min}\right\}$, the coverage $R(s_{i})$ includes no more than α PoIs; thus, it can be covered by α copies of the sensor with velocity 0. According to the Pigeonhole principle, at least one section of $R(s_{i})$ with summed weight not less than $w(s_{i})/\alpha$ is covered by one sensor $s_{i}^{\prime }$, where $\omega (s_{i})$ is the sum of weight of PoIs on range $R(s_{i})$. Assigning this sensor $s_{i}^{\prime }$ for $R(s_{i})$ yields the approximation ratio $\frac{1}{\alpha }$ for Algorithm 1, and hence $\frac{1}{2\alpha }$-approximation for MPSCL by Theorem 3. The shift to avoid overlapping in the last past of Algorithm 2 would not reduce the performance guarantee. So, Algorithm 2 is $\frac{1}{2\alpha }$-approximation for MPSCL.    □

Since Algorithm 2 calls Algorithm 1, its time complexity is $O(N*{\left(M/K+1\right)}^{K})$, where $K=\lfloor log_{\alpha }\left(v_{\max}/v_{d}\right)\rfloor +2$ and integer α≥2 is a tradeoff between time complexity and performance ratio.

#### 4.3.2. Linear-Programming Solution with Randomized Rounding

The above rounding technique gives a polynomial-time solution with approximation ratio capped by $\frac{1}{4}$ (when α=2). In this subsection, we apply linear programming relaxation and randomized rounding technique to improve the approximation ratio to $\frac{1}{2}\left(1-1/e\right)\approx 0.31606$.

We use the following integer program (IP) to formulate the optimal separation strategy of MPSCL and get its (1−1/e)-approximation algorithm, thus achieving $\frac{1}{2}\left(1-1/e\right)$-approximation of the optimal solution to MPSCL according to Theorem 3. In the integer program, variable $z_{l}$ indicates whether the PoI *l* is covered (1 or 0). $\omega_{l}$ is the weight of the PoI *l*. $P_{ij}$ indicates the set of PoIs covered by sensor $s_{i}$ when it sweep covers the coordinate interval $\left[x_{j},x_{j}+v_{i}T/2\right]$, where $x_{j}$ is the coordinate of PoI *j*. Variable $y_{ij}$ indicates whether the set $P_{ij}$ is covered; namely, $y_{i}j=1$ indicates that the sensor *i* is deployed to the coordinate interval $\left[x_{j},x_{j}+v_{i}T/2\right]$. Constraint (5) means that, for each PoI *l* covered $\left(z_{l}=1\right)$, at least one set $P_{ij}$ of PoIs containing the PoI *l* must be selected, and no set containing the PoI *l* is included otherwise $\left(z_{l}=0\right)$. Constraint (6) means one sensor can be used only once.

$$\begin{array}{cc} & \max\sum_{l=1}^{n}\omega_{l}z_{l} \end{array}$$

(5) $$\begin{array}{ccc} s.t. & \sum_{\left(i,j\right):l\in P_{ij}}y_{ij}\geq z_{l} & \forall l\in [1,N], \end{array}$$

(6) $$\begin{array}{ccc} & \sum_{j}y_{ij}\leq 1 & \forall i\in [1,M]\\ & y_{ij}\in \left\{0,1\right\} & \forall i\in [1,M],j\in [1,N]\\ & z_{l}\in \left\{0,1\right\} & \forall l\in [1,N]. \end{array}$$

Replacing the integer constraints $y_{ij}\in \left\{0,1\right\}$ and $z_{l}\in \left\{0,1\right\}$ with $0\leq y_{ij}\leq 1$ and $z_{l}\leq 1$ relaxes the above integer program (IP) to the following linear program (LP):

$$\begin{array}{ccc} & \max\sum_{l=1}^{n}\omega_{l}z_{l}\\ s.t. & \sum_{\left(i,j\right):l\in P_{ij}}y_{ij}\geq z_{l} & \forall l\in [1,N]\\ & \sum_{j}y_{ij}\leq 1 & \forall i\in [1,M]\\ & 0\leq y_{ij}\leq 1 & \forall i\in [1,M],j\in [1,N]\\ & z_{l}\leq 1 & \forall l\in [1,N]. \end{array}$$

Let $\left(y^{*},z^{*}\right)$ be the optimal solution to the linear program. We apply randomized rounding to make sensor *i* to cover the set $P_{ij}$ with probability $y_{ij}^{*}$ independently, i.e., we set $y_{ij}=1$ with probability $y_{ij}^{*}$. That yields a randomized (1−1/e)-approximation algorithm for the optimal separation strategy of MPSCL, shown in Algorithm 3. We prove its performance ratio in Theorem 6. Then, using the method of conditional expectations to derandomize Algorithm 3, we can obtain a deterministic approximation algorithm, Algorithm 4, with the same performance ratio. According to Theorem 3, Algorithm 3 and Algorithm 4 are the randomized $\frac{1}{2}\left(1-1/e\right)$-approximation and the deterministic $\frac{1}{2}\left(1-1/e\right)$-approximation algorithm for MPSCL.
**Algorithm 3** MPSCL-Random**Input:** A set of sensors S with velocities $V=\{v_{1},v_{2},\ldots ,v_{M}\}$, A set of PoI P with locations $X=\{x_{1},x_{2},\ldots ,x_{N}\}$ and weight $W=\{\omega_{1},\omega_{2},\ldots ,\omega_{N}\}$, sweep period *T*.
**Output:** The sweep covered set $P_{S}$ of PoIs.
1: **for**
i←1 to *M*
**do**
2: **for**
j←1 to *N*
**do**
3:  Let $P_{ij}$ be the set of PoIs located in coordinate interval $\left[x_{j},x_{j}+v_{i}T/2\right]$;
4: **end for**
5: **end for**
6: compute an optimal solution $\left(y^{*},z^{*}\right)$ to the linear programming relaxation (LP);
7: **for**
i←1 to *M*
**do**
8: Make sensor $s_{i}$ cover from the PoI *j* independently with probability $y_{ij}^{*}$, the  set of covered PoIs is $P_{s_{i}}$;
9: **end for**
10: $P_{S}={⋃}_{i=1}^{M}P_{s_{i}}$;
11: **return**
$P_{S}$

**Theorem** **6.** *Algorithm 3 is a randomized $\frac{1}{2}\left(1-1/e\right)$-approximation algorithm for MPSCL.*

**Proof.**  The proof is similar to the proof of Theorem 5.10 in Reference [32]. In Algorithm 3, the fractional value $y_{ij}^{*}$ is interpreted as the probability that $P_{ij}$ is chosen. Let random variable $Z_{l}=1$ if the PoI *l* is covered (1≤l≤N); $Z_{l}=0$ otherwise. Then, the probability that the PoI *l* is not covered is the probability that all the sets including the PoI *l* are not chosen:

$$\begin{array}{cc} Pr[Z_{l}=0] & =\prod_{\left(i,j\right):l\in P_{ij}}\left(1-y_{ij}^{*}\right)\\ \; & \leq {\left[\frac{1}{n_{l}}\sum_{\left(i,j\right):l\in P_{ij}}\left(1-y_{ij}^{*}\right)\right]}^{n_{l}}\\ \; & ={\left(1-\frac{\sum_{\left(i,j\right):l\in P_{ij}}y_{ij}^{*}}{n_{l}}\right)}^{n_{l}}\\ \; & \leq {\left(1-\frac{z_{l}^{*}}{n_{l}}\right)}^{n_{l}}, \end{array}$$

where $n_{l}$ indicates the number of sets in which the *l*th PoI is included, the second inequality follows from Arithmetic-geometric mean inequality, and the last inequality follows from Constraint (5).

When k≥1, the function $f_{k}\left(x\right)=1-{\left(1-\frac{x}{k}\right)}^{k}$(0≤x≤1) is concave. So, the probability that the PoI *l* is covered is

$$\begin{array}{cc} Pr[Z_{l}=1] & \geq 1-{\left(1-\frac{z_{l}^{*}}{n_{l}}\right)}^{n_{l}}\\ \; & \geq \left(f_{n_{l}}\left(1\right)-f_{n_{l}}\left(0\right)\right)\times z_{l}^{*}+f_{n_{l}}\left(0\right)\\ \; & =\left[1-{\left(1-\frac{1}{n_{l}}\right)}^{n_{l}}\right]z_{l}^{*}. \end{array}$$

Let *W* be a random variable of the summed weight of the covered PoIs, and let $OPT_{LP}$ and $OPT_{IP}$ be the optimal value of the linear program (LP) and the integer program (IP), respectively. The expected value of the summed weight is:

$$\begin{array}{cc} E[W] & =\sum_{l=1}^{n}\omega_{l}E\left[Z_{l}\right]\\ \; & =\sum_{l=1}^{n}\omega_{l}Pr\left(Z_{l}=1\right)\\ \; & \geq \sum_{l=1}^{n}\omega_{l}z_{l}^{*}\left[1-{\left(1-\frac{1}{n_{l}}\right)}^{n_{l}}\right]\\ \; & \geq min_{k\geq 1}\left[1-{\left(1-\frac{1}{k}\right)}^{k}\right]\sum_{l=1}^{n}\omega_{l}z_{l}^{*}\\ \; & \geq \left(1-\frac{1}{e}\right)OPT_{LP}\\ \; & \geq \left(1-\frac{1}{e}\right)OPT_{IP}. \end{array}$$

In Algorithm 3, there is only one linear program needed to solve, so it is a polynomial-time algorithm. Now, we have proven that Algorithm 3 is a randomized (1−1/e)-approximation algorithm for the optimal separation strategy of MPSCL. According to Theorem 3, Algorithm 3 is a randomized $\frac{1}{2}\left(1-1/e\right)$-approximation algorithm for MPSCL.    □

Now, we show how to use the method of conditional expectations to derandomize Algorithm 3 to obtain Algorithm 4. In Algorithm 4, let random variable $Y_{ij}=1$ denote that sensor *i* covers the set $P_{ij}$, i.e., for sensor $s_{i}$, $y_{ij}=1,y_{i\bar{j}}=0$, where $\bar{j}=\left\{j^{\prime }\in \left[1,N\right],j^{\prime }\neq j\right\}$ and $y_{i\bar{j}}=0$ denote that $y_{ij^{\prime }}=0,\forall j^{\prime }\in \bar{j}$. Then, $Pr\left[Y_{ij}=1\right]=y_{ij}^{*}$. In *h*th interation, $Y_{h-1}=\left\{y_{ij}|i\in \left[1,h-1\right],j\in \left[1,N\right]\right\}$ is fixed. Set $y_{hj_{h}}=1$ to let the current conditional expectation maximized, i.e., $j_{h}=argmax_{j\in [1,N]}E\left[W|Y_{hj}=1;Y_{h-1}\right]$. After *M* iterations, all $y_{ij}$ for i∈[1,M], j∈[1,N] are set. We can get a deterministic solution with the same approximation ratio to that of Algorithm 3. The output in Algorithm 4 is the union of the sets $P_{ij_{i}}$ for 1≤i≤M.
**Algorithm 4** MPSCL-Derandomized**Input:** A set of sensors S with velocities $V=\{v_{1},v_{2},\ldots ,v_{M}\}$, A set of PoI P with locations $X=\{x_{1},x_{2},\ldots ,x_{N}\}$ and weight $W=\{\omega_{1},\omega_{2},\ldots ,\omega_{N}\}$, sweep period *T*.
**Output:** The sweep covered set $P_{S}$ of PoIs.
1: **for**
i←1 to *M*
**do**
2: **for**
j←1 to *N*
**do**
3:  Let $P_{ij}$ be the set of PoIs located in coordinate interval $\left[x_{j},x_{j}+v_{i}T/2\right]$;
4: **end for**
5: **end for**
6: compute an optimal solution $\left(y^{*},z^{*}\right)$ to the linear programming relaxation (LP).
7: **for**
i←1 to *M*
**do**
8: set $j_{i}=argmax_{j\in [1,N]}E\left(W|Y_{ij}=1;Y_{i-1}\right)$;
9: make sensor $s_{i}$ to cover the set $P_{ij_{i}}$ of PoIs;
10: **end for**
11: $P_{S}={⋃}_{i=1}^{M}P_{ij_{i}}$;
12: **return**
$P_{S}$

**Theorem** **7.** *Algorithm 4 is a deterministic $\frac{1}{2}\left(1-1/e\right)$-approximation algorithm.*

**Proof.**  As the explanation above, we prove the theorem by induction. Without loss of generality, we assuming all the sensors will be occupied, i.e., $\sum_{j\in [1,N]}Pr\left[Y_{ij}=1\right]=\sum_{j\in [1,N]}y_{ij}=1\;\mathrm{for}\;i\in \left[1,M\right]$.

In the first step, we choose $j_{1}=argmax_{j}\left\{E\left[W|Y_{1j}=1\right]|1\leq j\leq N\right\}$ and set $y_{1j_{1}}=1$, $y_{1\bar{j_{1}}}=0$. By the definition of conditional expectations,

$$E\left[W\right]=\sum_{j=1}^{N}E\left[W|Y_{1j}=1\right]Pr\left[Y_{1j}=1\right]$$

and $\sum_{j=1}^{N}Pr\left[Y_{1j}=1\right]=\sum_{j=1}^{N}y_{1j}=1$, then

$$E\left[W|Y_{1j_{1}}=1\right]=max_{j}\left\{E\left[W|Y_{1j}=1\right]\right\}\geq E\left[W\right].$$

We assume that $E\left[W|Y_{h}\right]\geq E\left[W\right]$. In the (h+1)th step (1≤h<M), we choose $j_{h+1}=argmax_{j}\left\{E\left[W|Y_{h+1,j}=1;Y_{h}\right]\right\}$ and set $y_{h+1,j_{h+1}}=1$, $y_{h+1,\bar{j_{h+1}}}=0$. By the definition of conditional expectations,

$$E\left[W|Y_{h}\right]=\sum_{j=1}^{N}E\left[W|Y_{h+1,j}=1;Y_{h}\right]\times Pr\left[Y_{h+1,j}=1\right]$$

and $\sum_{j=1}^{N}Pr\left[Y_{h+1,j}=1\right]=\sum_{j=1}^{N}y_{h+1,j}=1$, then

$$\begin{array}{cc} \; & E[W|Y_{h+1,j_{h+1}}=1;Y_{h}]\\ \; & =max_{j}\left\{E\left[W|Y_{h+1,j}=1;Y_{h}\right]\right\}\\ \; & \geq E[W|Y_{h}]\\ \; & \geq E[W]. \end{array}$$

After *M* iterations, all $\left\{y_{ij}|i\in \left[1,M\right],j\in \left[1,N\right]\right\}$ are set. We get a deterministic solution $W_{d}$ satisfying

$$W_{d}\geq E\left[W\right]\geq \left(1-1/e\right)OPT_{IP}.$$

Since it takes polynomial time to solve the linear programming formulation, and there are M×N linear programming formulations need to be solved, Algorithm 4 runs in polynomial time.

According to Theorem 3, Algorithm 4 is a deterministic $\frac{1}{2}\left(1-1/e\right)$-approximation algorithm. □

## 5. Simulation Experiments

In this section, simulation experiments are conducted by using MATLAB to compare the algorithms including MPSCL-Velocity-Rounding, MPSCL-Random, and MPSCL-Derandomized. Since all the studies so far have not given the optimal cooperation strategy, we discuss the approximation ratios of the above algorithms to the optimal separation strategy, which are proven in the above theorems. Theorems 5–7 have shown that the approximation ratios of the three algorithms to the optimal separation strategy are separately $\frac{1}{\alpha }$, 1−1/e, and 1−1/e. The optimal separation strategy can be obtained by solving IP with the toolbox “Yalmip” of MATLAB, which is a free optimization solution tool developed by Lofberg.

In the experiments, there are *N* PoIs randomly distributed on a line of length 500, in which sweep period is 1. The velocities of *M* sensors are uniformly, randomly generated at range $\left[v_{\min},v_{\max}\right)$. Set the parameter α=2. We let *N* vary from 200 to 1000, *M* vary from 5 to 30, $v_{\min}$ vary from 5 to $v_{\max}$, and $v_{\max}$ vary from 10 to 50. For each combination of network parameters, we randomly generate ten instances to obtain the average performance and the lower bound of the performance of each algorithm. In the experiment, none of the parameters showed a significant effect on the approximation ratio. As shown in Table 2, the performance lower bounds of the three algorithms all satisfy the theoretical analysis. Algorithm MPSCL-Random is a randomized approximation algorithm. It only needs to satisfy the algorithm performance at a high probability. Thus, it has a fluctuating performance, as shown in Table 2. However, it also shows better performance than Algorithm MPSCL-Velocity-Rounding. Algorithm MPSCL-Derandomized derandomized Algorithm MPSCL-Random, so it must have higher computational complexity and better performance than MPSCL-Random, as shown in Table 2.

**Figure 3:**
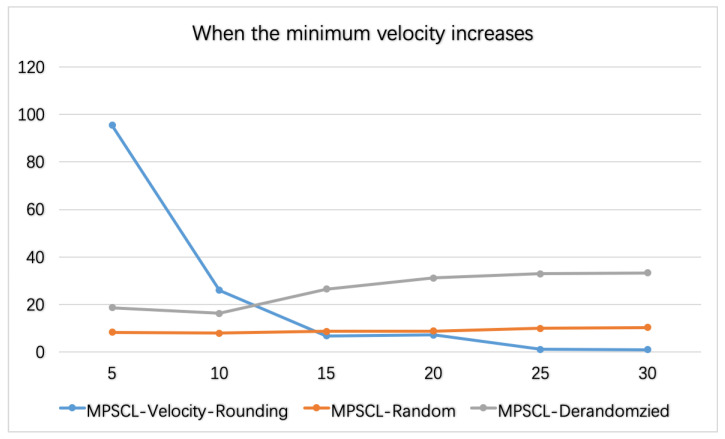
When the number of sensors remains the same, the influence of the minimum velocity of the sensors on running time. ($N=1000,M=20,v_{\max}=50$.)

**Figure 4:**
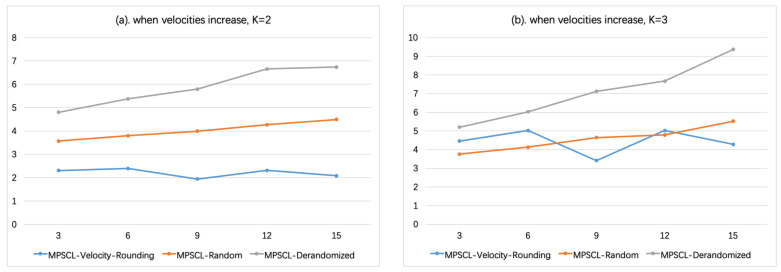
The influence of the velocities $\left[v_{\min},v_{\max}\right)$ on running time. (N=1000,M=10) (**a**). when $K=log_{2}\left(v_{\max}/v_{\min}\right)=2$. (**b**). when $K=log_{2}\left(v_{\max}/v_{\min}\right)=3$.

The influence of parameters on running time is shown below. Figure 3 shows the influence of the minimum velocity $v_{\min}$. In this experiment, the number of targets is N=1000, the number of sensors is M=20, the maximum velocity is fixed, $v_{\max}=50$, and the minimum velocity varies from 5 to 30. When the minimum velocity increases, the running time of Algorithm MPSCL-Velocity-Rounding decreases. Remind that the computational complexity of Algorithm MPSCL-Random is $O(N\times {\left(M/K+1\right)}^{K})$, where $K=\lfloor log_{\alpha }\left(v_{\max}/v_{d}\right)\rfloor +2$ and $v_{d}=\max\left\{\frac{1}{2T}d_{\min}^{\left(\alpha \right)},v_{\min}\right\}$. When α=2, the value of *K* is proportional to $v_{\max}/v_{\min}$. Therefore, when $v_{\min}$ increases, *K* decreases, which has a great impact on the complexity of Algorithm MPSCL-Velocity-Rounding. Thus, the running time becomes shorter. On the other hand, when the minimum velocity increases, the running time of Algorithm MPSCL-Random and MPSCL-Derandomized increased slightly. That is because, when $v_{\min}$ increases, so does the average velocity of the sensors, the coverage range of a sensor becomes larger, i.e., $|P_{ij}|$ in formula LP becomes larger, which makes the running time of Algorithm MPSCL-Random and MPSCL-Derandomized slightly increased. This can be shown more clearly in Figure 4. In Figure 4a,b, K=2 or K=3 is fixed, respectively, where *K* is the number of velocity groups. Let N=1000, M=10, $v_{\min}=$ 3:3:15. It is shown that the running time of Algorithm MPSCL-Velocity-Rounding would not be affected too much, and that of Algorithm MPSCL-Random, Algorithm MPSCL-Derandomzied would increase as $v_{\min}$ increases.

**Figure 5:**
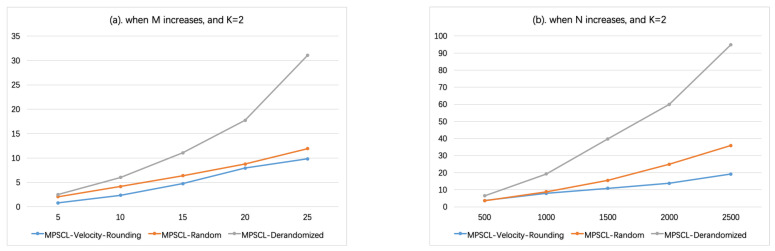
When K=2 ($v_{\min}=10$, $v_{\max}=40$). (**a**) The influence of the number of sensors *M* on running time. (**b**) The influence of the number of targets *N* on running time.

In Figure 5, set $v_{\min}=10$, $v_{\max}=40$, then the velocities of sensors can be divided into K=2 groups, [10,20),[20,40). Recall that the length of the line is 500. In Figure 5a, N=1000, the number of sensors *M* varies from 5 to 25. In Figure 5b, M=20, the number of targets *N* varies from 500 to 2500. Figure 5 shows that when K≤2, i.e., $v_{\max}/v_{\min}\leq 4$, the running time of Algorithm MPSCL-Velocity-Rounding is the shortest. That is reasonable because there is still no algorithm for solving linear programming with computational complexity less than $O(n^{2+\epsilon })$ (https://en.wikipedia.org/wiki/Linear_programming (accessed on 10 February 2021)).

Where *n* is the number of variables of linear programming, ϵ>0 is a fraction.

**Figure 6:**
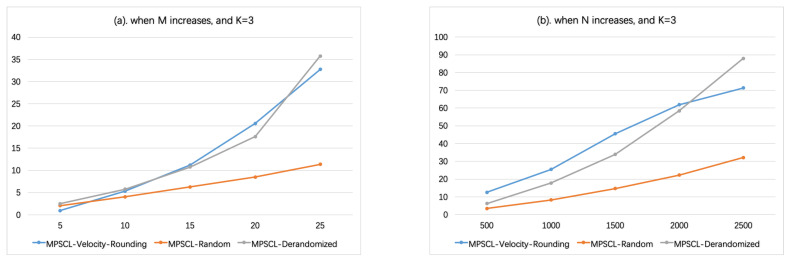
When K=3 ($v_{\min}=5$, $v_{\max}=40$). (**a**) The influence of the number of sensors *M* on running time. (**b**) The influence of the number of targets *N* on running time.

**Figure 7:**
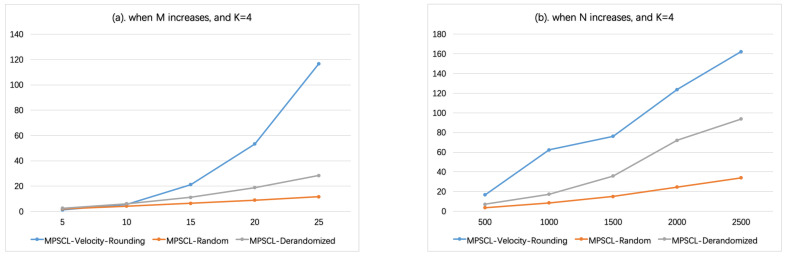
When K=4 ($v_{\min}=3$, $v_{\max}=48$). (**a**) The influence of the number of sensors *M* on running time. (**b**) The influence of the number of targets *N* on running time.

In Figure 6, set $v_{\min}=5$, $v_{\max}=40$; similarly, the velocities are divided into K=3 groups, [5,10),[10,20),[20,40). In Figure 6a, N=1000, the number of sensors *M* varies from 5 to 25. In Figure 6b, M=20, the number of targets *N* varies from 500 to 2500. There is the same parameter configuration except $v_{\min}=3$ and $v_{\max}=48$ in Figure 7, i.e., K=4. As shown in Figure 6 and Figure 7, when K≥3, the running time of Algorithm MPSCL-Random is shorter than Algorithm MPSCL-Velocity-Rounding. And as *K* or *M* increases, the running time of Algorithm MPSCL-Velocity-Rounding increases rapidly.

According to the experimental results, the performance and the computational complexity of the three algorithms all satisfy the theoretical analysis. Algorithm MPSCL-Random (Algorithm 3) shows excellent average performance. When $v_{\max}>4v_{\min}$, it would be a good choice if shortest running time is required. Or, if higher algorithm performance is asked for, Algorithm MPSCL-Derandomized (Algorithm 4) is better. When $v_{\max}\leq 4v_{\min}$, if the algorithm performance is acceptable, Algorithm MPSCL-Velocity-Rounding (Algorithm 2) can obtain faster results.

## 6. Conclusions

In this paper, we are the first to prove the PSCL problem is NPC by reducing 3-Partition problem to it and provide optimal and approximation algorithms for the MPSCL problem in different cases. For the special case when the velocities of the sensors are the same, we propose an optimal algorithm with a computational complexity of O(MN). For the case when the sensors have a constant number of different velocities, we use the dynamic programming method to find the optimal separation strategy and prove that it is a $\frac{1}{2}$ approximation algorithm for MPSCL. For the general case when the sensors have arbitrary velocities, we propose three approximation algorithms: one uses dynamic programming after velocity rounding to get an approximation ratio $\frac{1}{2\alpha }$; the second is a random approximation algorithm with an expected approximation ratio $\frac{1}{2}\left(1-1/e\right)$; and the third one derandomizes the random algorithm to get a deterministic algorithm with the same approximation ratio to the second one. All theoretical analyses are verified in experiments. Our future work is to study the Max-weighted Point Sweep Coverage problem in other types of graphs, such as trees and Eulerian graphs, and reduce the approximation ratio between the optimal value of MPSCL and that of the optimal separation strategy. Min-Sensor Sweep Coverage problem on Lines is also an interesting topic.

## Figures and Tables

**Table 1:** Notations.

Symbol	Definition
*N*	the number of the PoIs
*M*	the number of the sensors
P	the set of PoIs $\left\{p_{1},p_{2},..,p_{N}\right\}$
S	the set of mobile sensors $\left\{s_{1},s_{2},..,s_{M}\right\}$
X	the locations of PoIs $\left\{x_{1},x_{2},\ldots ,x_{N}\right\}$ $\left(x_{1}<x_{2}<\cdots <x_{N}\right)$
*T*	the sweep period of PoIs
V	velocities of the sensors $\left\{v_{1},v_{2},\ldots ,v_{M}\right\}$
W	the weight of the PoIs $\left\{\omega_{1},\omega_{2},\ldots ,\omega_{N}\right\}$
$P_{ij}$	the set of PoIs located on coordinate interval $\left[x_{j},x_{j}+Tv_{i}/2\right]$
$n_{ij}$	the number of PoIs in $P_{ij}$
$\omega_{ij}$	the summed weight of PoIs in $P_{ij}$
*W*	a random variable of the summed weight of the PoIs covered
$Y_{ij}$	random variables denote that sensor *i* covers the set $P_{ij}$
$v_{max}$	the largest velocity in V
$v_{\min}$	the smallest non-zero velocity in V

**Table 2:** The experimental performance of the algorithms.

Name of the Algorithm	Lower Bound of the Performance	Average Performance
MPSCL-Velocity-Rounding	0.66	0.81
MPSCL-Random	0.66	0.92
MPSCL-Derandomized	0.89	0.98
